# Bitter effect of a Sweet Disease‐ Exploring Cognitive dysfunction in Prediabetes from an Aging cohort

**DOI:** 10.1002/alz.091309

**Published:** 2025-01-09

**Authors:** Thomas Gregor Issac, Sandhya G, Anjana J Menon, Latha Diwakar, Jonas S. Sundarakumar

**Affiliations:** ^1^ Centre for Brain Research, Indian Institute of Science, Bangalore, Karnataka India; ^2^ Centre for Brain Research, Bangalore, karnataka India; ^3^ Centre for Brain Research, Bengaluru, Karnataka India; ^4^ Centre for Brain Research, Indian Institute of Science, Bengaluru, Karnataka India

## Abstract

**Background:**

India is unfortunately the “Diabetes Capital” of the world with estimated 101 million and 136 million patients suffering from diabetes and prediabetes respectively. Prediabetes is a transition state between euglycemia and diabetes. Although diabetes is associated with cognitive decline, studies that link prediabetes and cognition have been scarce and inconclusive especially from the Low and Middle Income (LMIC) countries. Therefore, the current study attempts to explore the relationship between prediabetes and cognition.

**Methods:**

531 participants aged ≥ 45 years, recruited by CBR‐TLSA (Tata Longitudinal Study of Ageing) were included in this study. Glycosylated Haemoglobin (HbA1c) levels was utilized to categorise the participants into healthy, prediabetes and diabetes based on the American Diabetes Association (ADA), 2017 criteria. Among these participants, 65 were healthy and 234 were prediabetic. The remaining 232 participants with Diabetes were excluded and directed towards appropriate care pathways. The COGNITO (Computerized Assessment of Adult Information Processing) neuropsychological test battery was utilized to assess the cognitive functioning. Generalized Linear Model (GLM) was performed between prediabetes status and cognition after adjusting for confounding variables.

**Results:**

The estimated prevalence of prediabetes in our elderly cohort is 44%. After adjusting for ApoE status, GLM analysis revealed that healthy participants performed better in semantic association (B = 0.314, 95% CI (0.047, 0.582), p = 0.021) and name‐face recognition (B = 1.644, 95% CI (0.516, 2.77), p = 0.004) tasks when compared to prediabetics. Subsequently, after adjusting for age, gender, years of education and ApoE status, it was observed that the scores of semantic association (B = 0.290, 95% CI (0.025, 0.556), p = 0.032) and name‐face recognition (B = 1.390, 95% CI (0.260, 2.520), p = 0.016) tasks were higher among healthy participants.

**Conclusion:**

This study emphasizes the deleterious effect of a potentially modifiable risk factor like prediabetes. Participants with prediabetes performed poorer in memory and language related cognitive tasks. If diagnosed early, interventions aimed at improving prediabetes could potentially prevent or delay the progression to both minor and major neurocognitive disorders.

